# Functionalized carbon nanoparticles for smartphone-based sensing of formaldehyde

**DOI:** 10.1039/d5na00865d

**Published:** 2025-11-26

**Authors:** Alessia Cavallaro, Lorenzo Russo, Víctor Sebastián, Roberta Ruffino, Giovanni Li Destri, Loredana Ferreri, Grazia Maria Letizia Consoli, Antonino Gulino, Angelo Ferlazzo, Andrea Pappalardo, Rossella Santonocito, Manuel Petroselli, Nunzio Tuccitto, Giuseppe Trusso Sfrazzetto

**Affiliations:** a Department of Chemical Sciences, University of Catania Viale A. Doria 6 95125 Catania Italy giuseppe.trusso@unict.it; b Instituto de Nanociencia y Materiales de Aragoń Aragón (INMA), CSIC-Universidad de Zaragoza, Campus Rio Ebro Edificio I + D + I, C/Poeta Mariano Esquillor, S/N 50018 Zaragoza Spain; c Department of Chemical and Environmental Engineering, Institute of Nanoscience and Materials of Aragon, Universidad de Zaragoza Zaragoza Spain; d Networking Research Center in Biomaterials, Bioengineering and Nanomedicine (CIBER-BBN), Instituto de Salud Carlos III 28029 Madrid Spain; e Laboratorio de Microscopías Avanzadas, Univ. de Zaragoza 50018 Zaragoza Spain; f CSGI Consorzio Interuniversitario per lo sviluppo dei Sistemi a Grande Interfase Via della Lastruccia 3 Firenze Italy; g Institute of Biomolecular Chemistry – CNR Via Paolo Gaifami 18 95126 Catania Italy; h Department of Science and Technological Innovation, University of Eastern Piedmont “Amedeo Avogadro” Viale Teresa Michel 11 15121 Alessandria Italy

## Abstract

Formaldehyde (FA) is a volatile organic compound of significant environmental and health concern due to its toxicity and widespread presence in indoor and industrial settings. The development of sensitive, selective, and user-friendly detection systems for FA is therefore of critical importance. In this work, we report a novel fluorescent nanosensor based on carbon nanoparticles functionalized with dopamine for the detection of FA in both aqueous and gaseous phases. The system achieved remarkable limits of detection—87 ppb in water and 10 ppb in air—well below the safety thresholds recommended by the World Health Organization. The sensing performance arises not only from the intrinsic photophysical properties of the carbon core but also from its architecture, which allows the anchoring of multiple recognition sites on a single nanoparticle. This multivalent interaction strategy increases the likelihood of FA binding events, enhancing both sensitivity and selectivity. Computational analysis supports the central role of the nanoparticle in the recognition process. The sensor operates effectively in solution and the solid state, and its compatibility with smartphone-based detection paves the way for the development of portable, low-cost devices for real-time FA monitoring.

## Abstract 2

A dopamine-functionalized carbon nanoparticle enables supramolecular, smartphone-based detection of formaldehyde in air and water, with limits of detection well below WHO guidelines.

## Graphical abstract

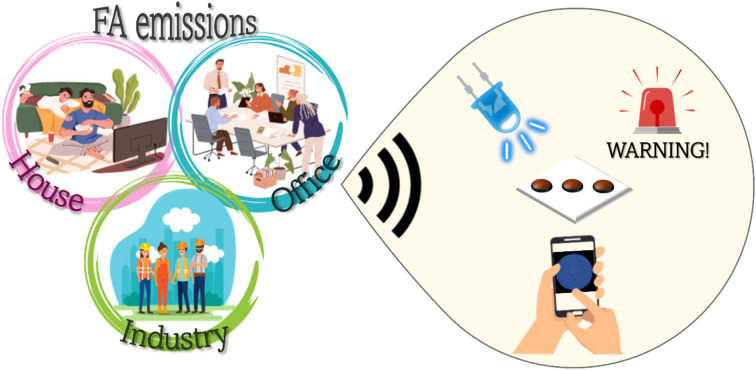

## Introduction

Formaldehyde (FA) is a volatile organic compound that appears as a colourless gas at room temperature, with a pungent odour. It is also flammable and highly reactive, so it can undergo several reactions in ambient air, such as photo-oxidation^1^ and radical reactions.^2^ FA is widely present in the environment, as it originates both from natural processes and human activities. Natural emissions arise from wildfires, vegetation decay and microbial processes.^3,4^ On the anthropogenic side, FA is released through industrial emissions, fuel combustion^5^ and cigarette smoke.^6^ Beyond these emissions, FA is also widely used in industry as an intermediate in the synthesis of chemicals and for the production of various materials, including resins,^7^ wood products,^8^ disinfectants^9^ and food preservatives.^10^ Due to its extensive application in building materials and furniture, FA is recognized as one of the major indoor air pollutants, as it is produced from the off gassing of these products over time.^11,12^ Consequently, FA levels in indoor air are often significantly higher than outdoors. This contamination is particularly worrisome given FA's toxicity. Inhalation of FA at concentrations between 120 ppb and 1 ppm can lead to a range of health issues, including eye and mucous membrane irritation, respiratory disfunctions and central nervous system disturbances.^13^ Long-term exposure has been associated with more severe outcomes, such as nasopharyngeal cancer and leukaemia.^14^ For these reasons, the World Health Organization (WHO) imposed that the safe limit on FA exposure in indoor air must not exceed 80 ppb over 30 minutes.^15^ This highlights the urgent need to develop sensors for FA detection, especially in the gas phase, capable to operate with high selectivity and sensitivity to monitor air quality in indoor environments.

Typically, FA detection is performed by diverse sensitive and selective instrumental methods, such as gas chromatography, often coupled with mass spectrometry.^16^ However, they represent a huge investment in terms of instruments, technology and skilled operators, making them impractical for point-of-need monitoring. Furthermore, the time required for a single analysis may be relevant and not useful to alert about the presence of FA. Electrochemical techniques are also employed for FA detection,^17^ providing cheaper tools to obtain fast responses with good sensitivity, but they suffer limited reliability in the presence of interferents, so that frequent calibrations are required.^18^ In this context, optical sensors have emerged as a powerful tool for the detection of FA, as they meet the demand for sensitive and selective detection, while remaining low cost and user-friendly. Among them, fluorescent sensors have been extensively employed in both solution and the gas phase, thanks to their high sensitivity and ease of signal transduction.^19^ Nevertheless, the simple reaction or interaction between a small-molecule probe and an analyte can often be limited by long reaction times and binding efficiency, especially at low concentrations, respectively. In this regard, nanostructured platforms offer significant advantages, such as a large surface area, multiple binding sites and tuneable surface functionalities, thus enhancing the probability and strength of interaction with the target analyte.^20^ In fact, it is not by chance that, currently, nanostructured sensing architectures, such as quantum dots (QDs),^21–24^ metal organic frameworks (MOFs)^25–27^ and macrocyclic systems,^28^ provide one of the most effective strategies for FA sensing, reaching the lowest limits of detection.^19^ In this context, fluorescent carbon nanoparticles (CNPs) have attracted increasing interest as a sustainable and versatile sensing platform, due to their exceptional synthetic and optical features.^29^ Synthesis is usually simple, including solvent-free carbonization,^30^ laser ablation,^31^ microwave irradiation^32,33^ and hydrothermal decomposition.^34^ Common carbon precursors are citric acid^35^ and polymers,^36^ and even food waste,^37^ therefore making the synthesis environmentally friendly. CNPs also possess remarkable optical properties. They show a characteristic absorption band due to the π–π* transition of sp^2^ carbons of the aromatic core, and an n–π* transition associated with the presence of heteroatoms (N, S, P, *etc.*). Their fluorescence emission ranges from 10% quantum yields up to 80%.^38^ A particular behaviour can be observed quite often, that is the dependence of the emission wavelength (*λ*_em_) on the excitation wavelength (*λ*_ex_).^39^ This behaviour can be modulated either by doping CNPs with heteroatoms or through surface modification. The latter feature is particularly advantageous: depending on the synthetic route, CNPs exhibit a variety of functional groups on their external shell, whose chemical reactivity can be exploited to tailor the surface properties, thus tuning solubility, emission and ability to recognize a target analyte.^29,40^

Literature analysis highlights that a copolymer sensor shows superior sensitivity detectable by the naked eye.^28^ In addition, smartphone-based covalent systems combine good sensitivity with user-friendly operation.^41^ Some supramolecular systems showed good results in terms of limits of detection (LODs) and the advantage of smartphone-based detection.^27,42,43^ These findings underline the effectiveness of both covalent and supramolecular approaches, with a growing emphasis on portable, accessible sensing technologies.

In this work, we present a novel optical sensor to detect FA both in aqueous and gaseous environments, based on carbon nanoparticles functionalized on their external shell with dopamine moieties as recognition sites (CNPs-DA). Following synthesis, functionalization, and extensive morphological and structural characterization of the CNPs-DA, their use as FA sensors was first tested in aqueous medium, where selectivity was also screened. Finally, we developed a solid-state sensor to assess the detection performances of CNPs-DA towards gaseous FA, reaching an experimental LOD of 10 ppb, significantly lower than the WHO guideline threshold. Notably, the solid-state sensor can be used in combination with a simple smartphone as the detector, thus obtaining a practical device for FA monitoring in the gas phase.

## Results and discussion

### Design, synthesis and characterization of the nanosensor (CNPs-DA)

The decision to functionalize CNPs with dopamine was driven by the aim of developing a fluorescent nanosensor with selective recognition capabilities for FA. The CNPs serve a dual role: they act as the fluorescent core, owing to their distinctive emission properties, and as a versatile scaffold for multivalent functionalization. Dopamine was chosen because it offers a preorganized recognition site capable of establishing two hydrogen bonds with the oxygen atom of FA (SI, Scheme S1). The resulting nanosensor enables the attachment of multiple dopamine units on a single nanostructure, thereby significantly enhancing sensitivity towards FA.

**Scheme 1:**
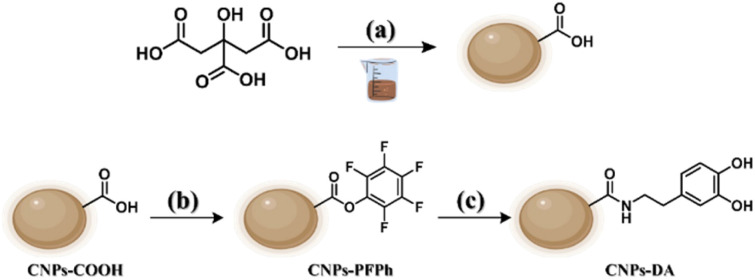
Reaction pathway for the obtaining of CNPs along with conditions: (a) 300 °C, NaOH_aq_ 0.1 M, 30 minutes. (b) Pentafluorophenol, EDC hydrochloride, 50 °C, N_2_, 48 h. (c) Dopamine hydrochloride, DIPEA, room temperature, CH_2_Cl_2_, N_2_, 4 days.

The obtaining of CNPs-DA involved three main steps, each corresponding to a distinct reaction. The first step was the synthesis of CNPs-COOH, using citric acid as a precursor *via* solvent-free carbonization. This process reached a temperature of 300 °C, producing a dark brown solid that was solubilized in NaOH aqueous solution. Purification was made through centrifugation to precipitate larger aggregates, and then dialysis to eliminate smaller molecules, therefore achieving a batch of morphologically uniform CNPs. Due to the chemical structure of citric acid, most of the functional groups on the outer shell of the resulting nanoparticles are carboxylic acids, whose reactivity can be exploited for further functionalization (Scheme 1a). The second step took advantage of this reactivity, consisting in the activation of carboxylic groups with pentafluorophenol (PFPh), in presence of a carbodiimide as a coupling agent (Scheme 1b). The reaction was carried out by solvolysis in PFPh. This step was crucial because the resulting CNPs-PFPh were soluble in CH_2_Cl_2_, allowing the following nucleophilic substitution that cannot be performed in water.

The final step was the functionalization of CNPs-PFPh with dopamine, using DIPEA as a base to increase the nucleophilicity of the aminic group (Scheme 1c). Then, their morphology, chemical structure and composition were investigated, as well as their optical properties.

#### Morphological characterization (AFM and TEM-EDS)

**Fig. 1:**
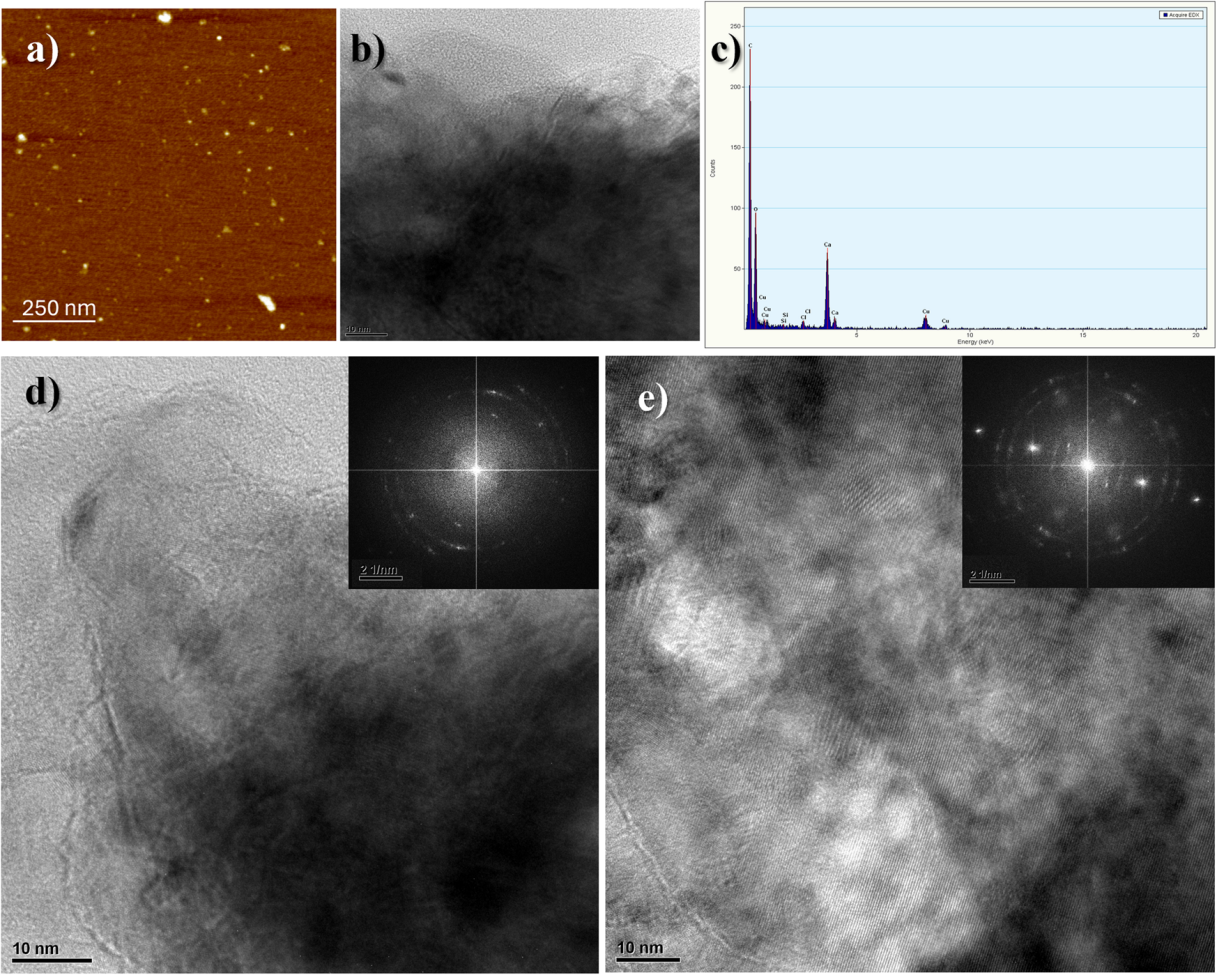
(a) AFM image of the CNPs-DA. (b) TEM image of CNPs-DA, revealing the laminar structure and tendency to aggregate. (c) EDS spectrum confirming a predominant composition of carbon and oxygen, with trace amounts of calcium (Ca) and chlorine (Cl). (d and e) Representative HRTEM images of a CNP displaying partially graphitized regions; the insets show the corresponding FFT.

Atomic force microscopy (AFM) characterization (Fig. 1a) revealed the expected disk-like morphology with narrow dispersity, as CNPs-DA have a diameter of 22 ± 5 nm and a height of 2 ± 1 nm.

High-resolution transmission electron microscopy (HRTEM) and energy-dispersive X-ray spectroscopy (EDS) were employed to further characterize the CNPs. These CNPs exhibit a turbostratic graphitic structure, defined by graphene-like layers that are stacked without long-range order, resulting in a mixture of crystalline and amorphous features, as clearly observed in the HRTEM images.^44^ The images reveal the formation of ultrathin carbon sheets (Fig. 1b) that are in agreement with AFM analysis (Fig. 1a) and which assemble into more complex architectures. These nanostructures display coexisting graphitic and amorphous domains, with the amorphous carbon preferentially located at the outer regions of the particles, forming a disordered shell around a more crystalline core. EDS analysis confirms a predominant composition of carbon and oxygen, in agreement with the carbonization of citric acid, a process known to introduce oxygen-rich functional groups (Fig. 1c). Additionally, trace amounts of calcium (Ca) and chlorine (Cl) are detected, attributed to residual ions due to the dialysis step. At the structural level, representative HRTEM imaging (Fig. 1d and e) shows well-defined lattice fringes corresponding to graphitic domains, interspersed with amorphous regions. In several areas, turbostratic carbon is observed, an intermediate form of carbon characterized by misaligned graphene-like layers and disordered stacking lacking long-range periodicity. These regions typically contain a mixture of sp^2^- and sp^3^-hybridized carbon,^44^ reflecting the structural complexity of the material. Fig. 1d shows a representative HRTEM image where the FFT reveals *d*-spacings of 0.244 and 0.342 nm, corresponding to in-plane graphitic ordering and an expanded (002) reflection, respectively. The latter is characteristic of turbostratic stacking, where the layers are randomly oriented relative to each other, leading to increased interlayer distances. These features are consistent with the structural outcome expected from pyrolyzed citric acid, which tends to form partially graphitized carbon with misoriented layers. In Fig. 1e, the HRTEM image displays a polycrystalline morphology, with multiple nanocrystalline domains oriented randomly. The corresponding FFT shows several concentric diffraction rings with *d*-spacings of 0.198, 0.245, 0.293 and 0.386 nm. The reflections in the 0.198–0.245 nm range are consistent with in-plane graphitic planes, such as the (100) and (110). The peak at ∼0.386 nm corresponds to the (002) reflection of turbostratic graphite, typically broadened and shifted to higher *d*-values due to stacking disorder and increased interlayer spacing. Altogether, the FFT pattern, with concentric rings and broadened features, confirms the presence of randomly oriented crystalline domains embedded in a disordered carbon matrix, supporting the polycrystalline and heterogeneous nature of the CNPs.

#### Chemical characterization (NMR, XPS and FT-IR)

In order to understand if the functionalization occurred, chemical characterization of CNPs-DA was performed through various techniques. The ^1^H NMR spectrum of CNPs-DA, recorded in DMSO-*d*_6_, confirms the presence of all the protons belonging to the dopamine moiety. The aliphatic protons of the two methylene groups show a downfield shift relative to the starting free dopamine,^45^ consistent with amide bond formation and thus suggesting successful functionalization of nanoparticles (SI, Fig. S1).

**Fig. 2:**
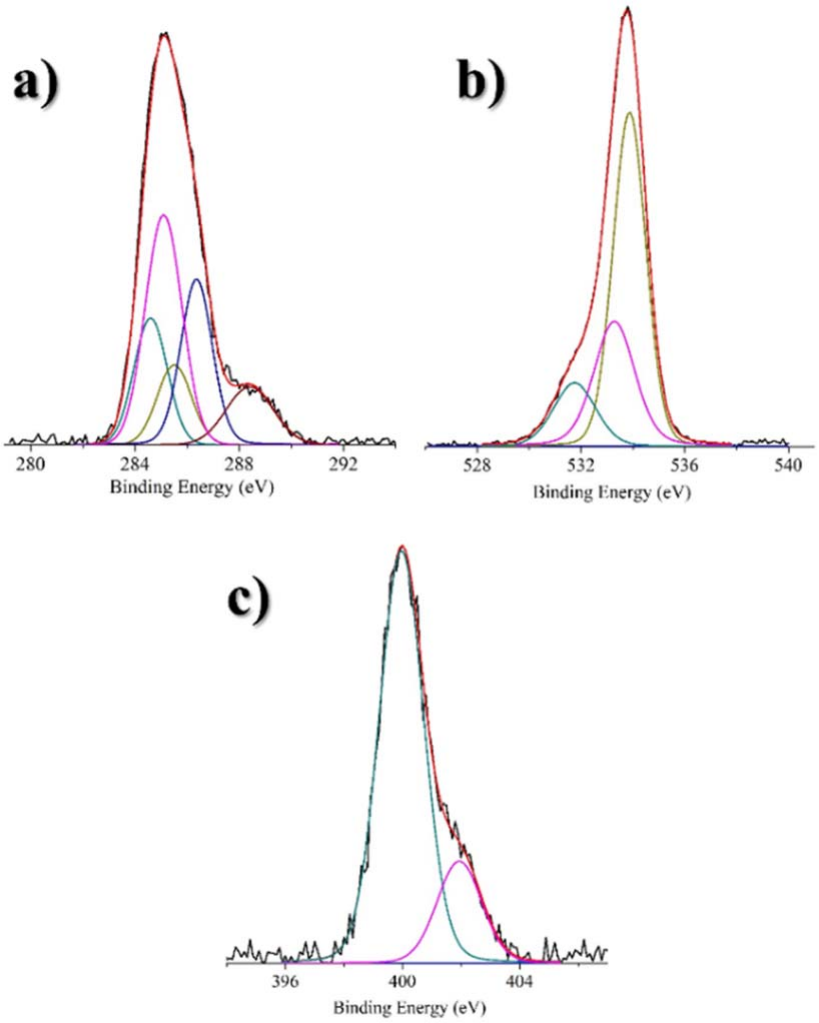
In the three spectra, the blue line represents the background, and the red line superimposed to the experimental black profile refers to the sum of the Gaussian components. (a) Al Kα excited XPS of CNPs-DA in the C 1s binding energy region: the dark cyan, magenta, dark yellow, navy, and wine lines refer to the 284.6, 285.0, 285.5, 286.4, and 288.4 eV Gaussian components, respectively. (b) Al Kα excited XPS of CNPs-DA in the O 1s binding energy region: the dark cyan, magenta, and dark yellow lines refer to the 531.8, 533.3 and 533.9 eV Gaussian components, respectively. (c) Al Kα excited XPS of the CNPs-DA in the N 1s binding energy region: the dark cyan, and magenta lines refer to the 400.0, and 401.9 eV Gaussian components, respectively.

X-ray photoelectron spectroscopy (XPS) was performed to further investigate the electronic structure of CNPs and verify the presence of dopamine moieties on the nanoparticle surface. Fig. 2a shows the XP spectrum of CNPs-DA in the C 1s binding energy region. Two experimental peaks at 285.2 and 288.3 eV are evident. A careful deconvolution of the experimental spectrum required five Gaussians at 284.6 eV due to the C sp^2^ states, 285.0 eV due to the C sp^3^ states (C–C and C–H) and some adventitious carbon, 285.5 eV due to the C–N levels, 286.4 eV due to the C–OH states, and 288.4 eV due to the O=C–N amide group.^46,47^ The intensity ratio of the C sp^2^/C sp^3^/C–N/C–OH/O=C–N bands is 1.5/3/1/2/1. In Fig. 2b, the XP spectrum of CNPs-DA in the O 1s binding energy region is reported. The fitting required three Gaussians at 531.8 eV due to the O=C–N states, 533.3 eV due to the C–OH levels, and 533.9 eV due to the oxygen of the substrate (SiO_2_).^47^ The intensity ratio of the first two bands (O=C–N/C–OH) is 1/2, in agreement with the structure of the nanoparticles (Scheme 1). Finally, in Fig. 2c, the N 1s binding energy region is shown. Two experimental peaks at 400.0 and 402.0 eV are evident. A careful deconvolution of the experimental spectrum required two Gaussians at 400.0 eV due to the N–C=O amide states and 401.9 eV due to some quaternized nitrogen^48,49^ (probably due to the amide protonation), which does not affect the sensing performances. The relative intensity percentage of N–C=O/N^+^ is 80/20. A table with XPS binding energies and assignment is reported in the SI (Table S1).

FT-IR characterization was performed to support the evidence of successful functionalization. A comparison between the FT-IR spectra of CNPs-PFPh and CNPs-DA shows the disappearance of the bands at 996 and 1009 cm^−1^, attributed to the C–F stretching of pentafluorophenol (SI, Fig. S2). The functionalization of the amide is further confirmed by the presence of the band at 1635 cm^−1^, typical of the C=O stretching of the secondary amide.^50^ This spectral change provides a relevant contribution to the overall characterization of the functionalization process.

#### Optical characterization (UV-visible and fluorescence)

**Fig. 3:**
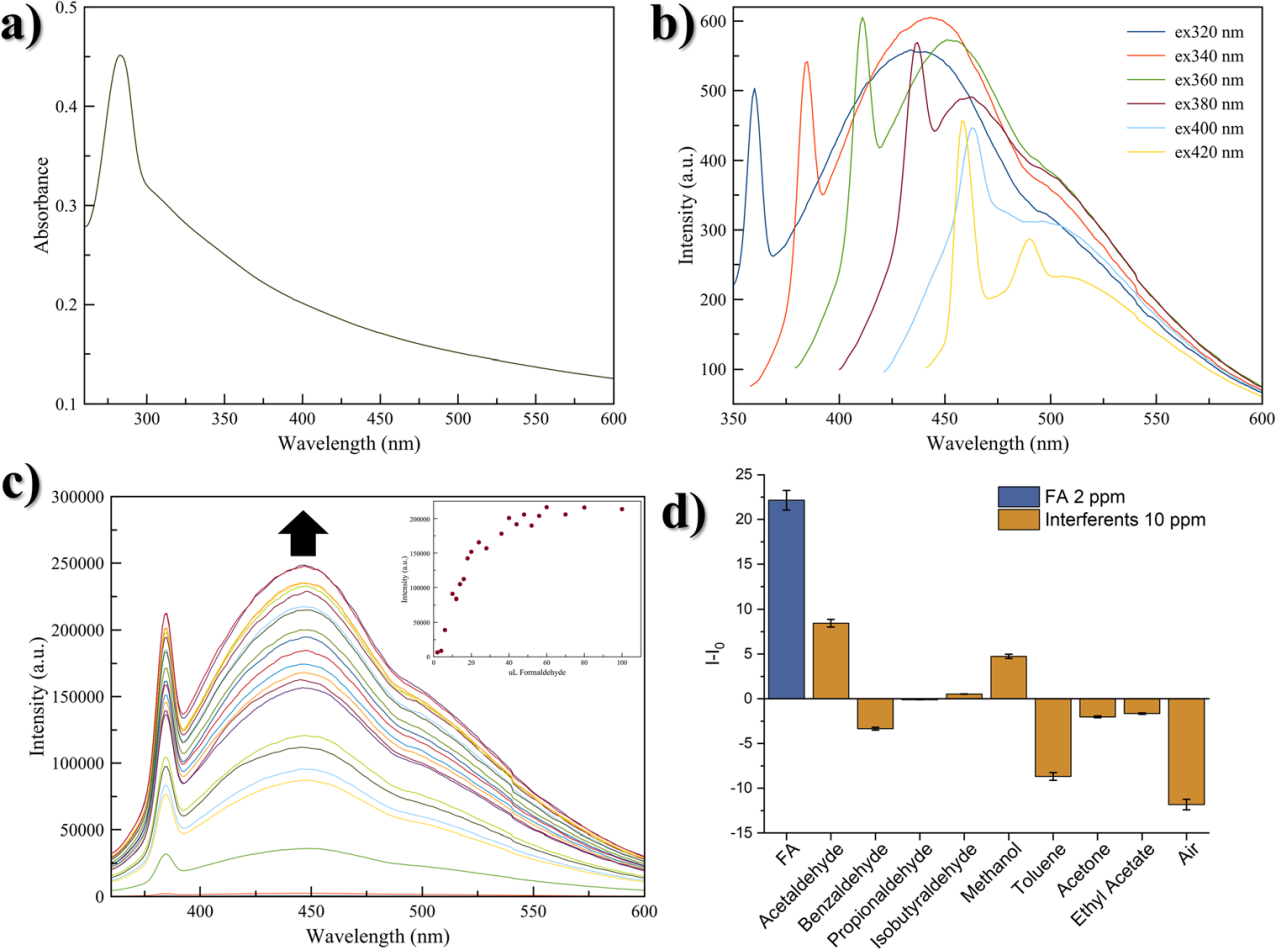
(a) UV-visible spectrum of CNPs-DA at concentration of 0.1 mg mL^−1^ in water. (b) Emission spectra of CNPs-DA at different *λ*_ex_ (from 320 nm to 420 nm, see the inset of the figure), at concentration of 0.1 mg mL^−1^ in water. (c) Normalized emission spectra of CNPs-DA (0.1 mg mL^−1^ in MilliQ water, *λ*_ex_ = 340 nm) upon addition of progressive amount of FA, in concentration range 0.1–5 ppm. The inset shows the titration curve. (d) Selectivity tests: the histogram is plotted as the difference Δ*I* = *I* − *I*_0_, where *I* is the fluorescence intensity of CNPs-DA in the presence of the interferent, and *I*_0_ is the fluorescence intensity associated with CNPs-DA in MilliQ water. Error bars represent the standard deviation associated with the replicates.

The optical features of the synthesized CNPs were investigated using UV-vis and fluorescence measurements. The UV-vis spectrum of CNPs-DA (0.1 mg mL^−1^ in water) shows a band at 283 nm, corresponding to dopamine absorption in neutral aqueous solution.^51^ Moreover, the CNPs contribute to the experimentally observed broad band, which can be ascribed to π–π* and n–π* charge transfer transitions of the conjugated C=C framework occurring at around 300 nm (Fig. 3a). In fact, due to the weak interaction between the layers and the ease of accessing low energy conformations, graphitic CNPs tend to exhibit significant structural disorder, which leads to broadening of the bands.^52^

Fluorescence spectra of the synthesized CNPs were recorded in water at a concentration of 0.1 mg mL^−1^. The emission profiles display the typical excitation-wavelength dependence commonly observed in carbon-based nanostructures.^37^ As shown in Fig. 3b, along with the variation of *λ*_ex_ from 320 nm to 420 nm, a progressive red-shift of the emission maximum can be noticed, ranging from 434 nm to 475 nm. A shoulder centred at 497 nm is detected, remaining constant across the excitation range. Given its excitation-independent behaviour, this signal is likely not related to the emissive core of the nanoparticle, but instead to the surface functionalization.^53^ Additionally, at *λ*_em_ lower than the emission maximum, a sharp band is visible, shifting accordingly with *λ*_ex_. This property is attributed to the Raman scattering of water, a well-known phenomenon in fluorescence measurements.^54^

### FA sensing in solution

#### CNPs-DA fluorescence titration with FA

The recognition ability of CNPs-DA towards FA was first evaluated in aqueous solution. To this aim, a solution of CNPs-DA (0.1 mg mL^−1^ in MilliQ water) was placed in a quartz cuvette and titrated with increasing volumes of a FA aqueous solution at a concentration of 10^−4^ M, ranging from 2 to 100 µL, corresponding to a FA concentration range from 0.1 to 5 ppm in the cuvette. As FA concentration increases, a gradual enhancement in the emission of CNPs-DA can be observed (Fig. 3c). An increase of 12.5% of the quantum yield was observed upon addition of 5 ppm of FA.

The strong interaction between FA and CNPs-DA is confirmed by the high binding constant, reaching log *β* = 5.87 ± 0.08.††The standard deviation considers the uncertainties on the CNPs' dimensions and the possibility of lower surface coverage with dopamine. The limit of detection (LOD), calculated using the formula LOD = 3*σ*/*k*, is 87 ppb.

This value is one order of magnitude lower than the limit of 900 ppb set by the WHO for drinking water, suggesting the suitability of the nanosensor for FA detection in water.^55^

#### Selectivity tests

**Fig. 4:**
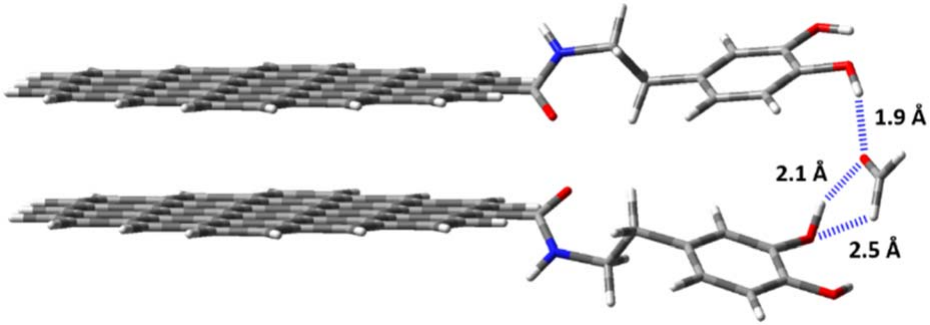
Optimized structure of the host–guest (HG) complex between the dopamine-functionalized material (CNPs-DA) and FA. Non-covalent interactions between the host and the guest, mainly hydrogen bonds (HBs), are marked with a dashed blue line. HB lengths are reported in angstrom (Å).

Evaluating selectivity is essential to ensure reliable detection, both against structurally similar compounds and against substances typically present in the environment where the target analyte is detected. To this end, selectivity tests were performed in aqueous solution, assessing CNPs-DA's response to an excess of each interferent with respect to FA (Fig. 3d). The selected interferents belong to two families of compounds: low-molecular weight aldehydes, such as acetaldehyde, propionaldehyde, iso-butyraldehyde and benzaldehyde, and common volatile organic compounds (VOCs), such as methanol, acetone, ethyl acetate and toluene. Moreover, air, containing 24 000 ppm of water, 400 ppm CO_2_, 5 ppm NO, and 10 ppm CO, was tested as a potential interferent by gently bubbling it into the cuvette. Fig. 4d shows the emission response of CNPs-DA to these analytes, confirming the excellent selectivity towards FA (detailed emission spectra are reported in the SI, Fig. S3a–i).

### Sensing mechanism

To further elucidate the sensing mechanism and selectivity of CNPs-DA towards FA, a DFT study was performed. Two graphene surfaces (1.2 × 1.1 nm), positioned 0.3 nm apart, were used as a model system for the CNP core (Fig. S4). The borders of the CNPs were functionalized with two dopamine units to simulate the CNPs-DA material. It is worth noting that the proposed model CNPs-DA system was designed based on the characterization data experimentally obtained (*vide supra*).

A conformational study on CNPs-DA revealed the accessible orientations of the two dopamine moieties. Specifically, the main factor influencing the conformation is the amide group(s), which can adopt configurations where the carbonyl groups either face each other (Fig. S5 – conformation A) or lie parallel to each other (Fig. S5 – conformation B). In conformation B, the potential hydrogen bond between the amide N–H bond and the carbonyl group (C=O) is disfavoured due to the limited space between the two graphene layers (0.3 nm), which does not allow for proper orientations. Furthermore, the sterically hindered environment places electronegative atoms, such as oxygen and nitrogen, in close proximity, resulting in a strong repulsion and destabilizing the entire system. Indeed, the less hindered conformation A is more stable by around 8.30 kcal mol^−1^ (Fig. S5). The optimized CNPs-DA system was used to investigate its binding properties towards FA. The molecular electrostatic potential (MEP) map of FA reveals two distinct electron-density regions: (i) a partially negatively charged region on the carbonyl oxygen (C=O), and (ii) a partially positively charged region on the aldehyde hydrogens (C–H) (Fig. S6). This makes FA both a good hydrogen bond donor and acceptor, and an ideal match for dopamine derivatives such as CNPs-DA, due to the potential interactions with hydroxyl groups.^56^ Indeed, three hydrogen bonds are observed in the optimized FA@CNPs-DA complex: two classical hydrogen bonds between the hydroxyl group and the carbonyl oxygen (OH⋯O=C), and one CH hydrogen bond between the electropositive aldehyde hydrogen and the oxygen atom of the hydroxyl group (CH⋯OH) (Fig. 4).^57^ These non-covalent interactions ensure strong affinity between CNPs-DA and FA, as confirmed by the complexation energy (*E*_complex_) value of 11.9 kcal mol^−1^, calculated for the FA@CNPs-DA complex. This value aligns with the experimental value of log *β* = 5.87 ± 0.08, and the data reported in the literature for similar complexes, highlighting the reliability of the study.^37,58^ To further emphasize the high affinity between FA and CNPs-DA, we calculated the *E*_complex_ at the same level of theory for a water dimer (2H_2_O), finding a value of 5.9 kcal mol^−1^ – approximately half the value observed for the FA@CNPs-DA complex (Table S3). VOCs such as acetone and ethyl acetate, although containing a carbonyl group, exhibit lower affinity towards CNPs-DA due to the absence of hydrogen bond donors, which reduces the number of non-covalent interactions between the host and guest. This effect is extreme for toluene, which lacks both hydrogen bonding donors and acceptors, resulting in the worst guest for the CNPs-DA system. Instead, a higher affinity is observed for methanol (CH_3_OH), although it remains lower than that of FA, due to the absence of sufficiently electropositive hydrogen atoms capable of forming strong CH hydrogen bonds. Similarly, alkylated and more sterically hindered aldehydes (*e.g.*, propionaldehyde and benzaldehyde) exhibit lower affinity than FA primarily for two reasons: (i) longer and weaker hydrogen bonds resulting from the presence of bulkier groups than the hydrogen atom^57^ and (ii) less electropositive aldehydic hydrogen due to the electronic effect (*e.g.*, hyperconjugation) of the alkyl substituents on the carbonyl moiety.^59^

In order to support the hypothesis that both the covalent conjugation of nanoparticle and dopamine is responsible for the selective interaction with FA, further validation studies were carried out. In particular, we performed fluorescence titration between FA and CNPs-PEA (PEA = phenyl ethyl amine), which has a similar structure to CNPs-DA but lacks the two hydroxyl groups on the aromatic ring; these groups are supposed to be crucial for FA recognition. We also performed fluorescence titration between FA and *N*-acetyl-dopamine (which does not contain the nanoparticle moiety). In both cases, no recognition properties for FA were detected, supporting, together with DFT analysis, the synergy of the presence of carbon nanoparticles and the dopamine functional group in the detection of FA. The detailed synthetic procedures for the synthesis of CNPs-PEA and *N*-acetyl-dopamine are reported in the SI.

### Strip test for gaseous FA sensing

Then, we moved to the evaluation of the sensing performance of CNPs-DA in the solid state as a strip test to detect FA in the gas phase. A polyamide filter was chosen as the solid support, optimized in size to fit in the cap's inner part of a 20 mL vial. This configuration allows the sensor to be placed in contact with the environment of the vial once it is closed. Notably, this environment contains normal atmospheric air. On each strip test, three spots of CNPs-DA (0.5 mg mL^−1^ in MilliQ water) were drop casted. The selected method of detection was a commercial smartphone.^24,27,37,41,43,60–63^ The strip tests were irradiated with a UV LED at *λ*_ex_ = 365 nm in a 3D-printed dark chamber (Fig. S7), and images were taken with the smartphone before and after the exposure to gaseous FA.‡‡Images were acquired using three different smartphones (iPhone 13, iPhone 13 Pro and Samsung A22), all equipped with ProCam® application that, together with the dark chamber, allows eliminating the contribution of different ambient light sources. The full description of strip test preparation, sensing procedure and image processing can be found in the SI. Once drop casted onto the polyamide solid support, CNPs-DA show high stability over several days, as demonstrated in Fig. S9 (see the SI). Gas-phase FA detection experiments were based on literature reports that describe the solubility of gaseous FA in water and the generation of FA vapor from aqueous solutions, as a function of its vapor pressure at defined concentrations and temperature (see the Experimental section).^64^ Detailed calculations are reported in the SI.

#### Kinetics studies

To investigate the kinetics between the solid sensor and gaseous FA, nine vials were prepared: three containing only water and six containing aqueous FA solutions. The latter were designed to generate gaseous FA concentrations of 10 ppb and 1000 ppb at 25 °C, with three replicates each. First, an image of each strip test was taken with a smartphone. The nine vials were divided into three groups, each consisting of one vial containing water, one containing an aqueous FA solution generating 10 ppb of gaseous FA, and one containing an aqueous FA solution generating 1000 ppb of gaseous FA. The first group was analysed after 1 h, the second after 2 h, and the third after 3 h by acquiring another image of the solid sensor.

**Fig. 5:**
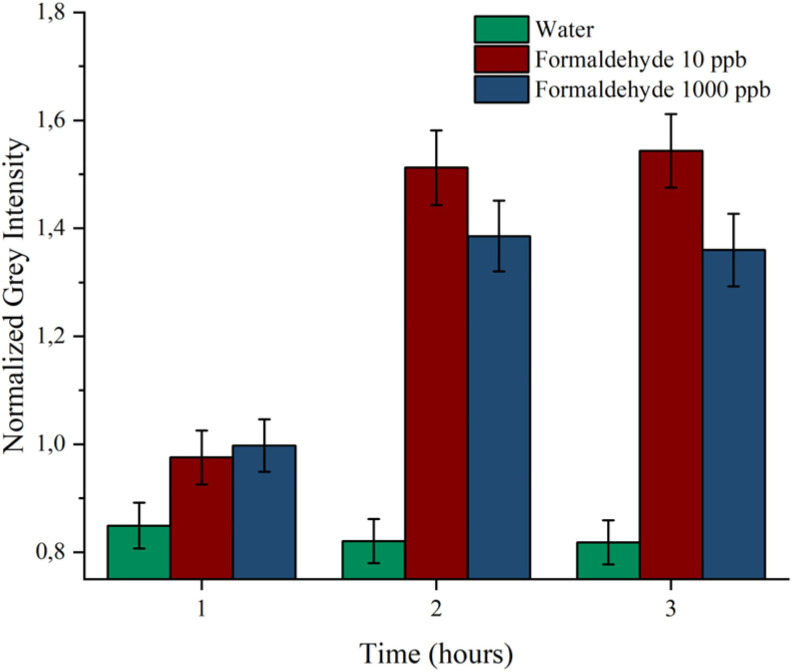
Histogram showing the normalized grey intensity for the sensor's exposure to water, 10 ppb and 1000 ppm of FA over the three hours of analysis, in green, red and blue, respectively. Error bars refer to the standard deviation of the replicates.

The image analysis revealed that after the first hour, both water and FA evaporation concurred to the variation of the fluorescence signal. However, after the second hour, a clear distinction emerged: the strip test exposed to FA solution showed a marked increase in intensity for both FA concentrations compared to the water control. This response remained unchanged after the second hour, indicating that the equilibrium is reached within 2 h of exposure (Fig. 5). Notably, the presence of water into the vials leads to the water vapour saturation that does not affect the response of the strip test.

In addition, the stability of the complex between CNPs-DA and 1 ppm of FA on the strip test was evaluated by measuring the optical response over 7 days (see Fig. S10 in the SI). In particular, the device shows a stable signal for 24 hours. Then, a progressive decrease of the emission can be observed, probably due to the non-covalent interaction involved in the FA sensing.

#### Calibration with gaseous FA

**Fig. 6:**
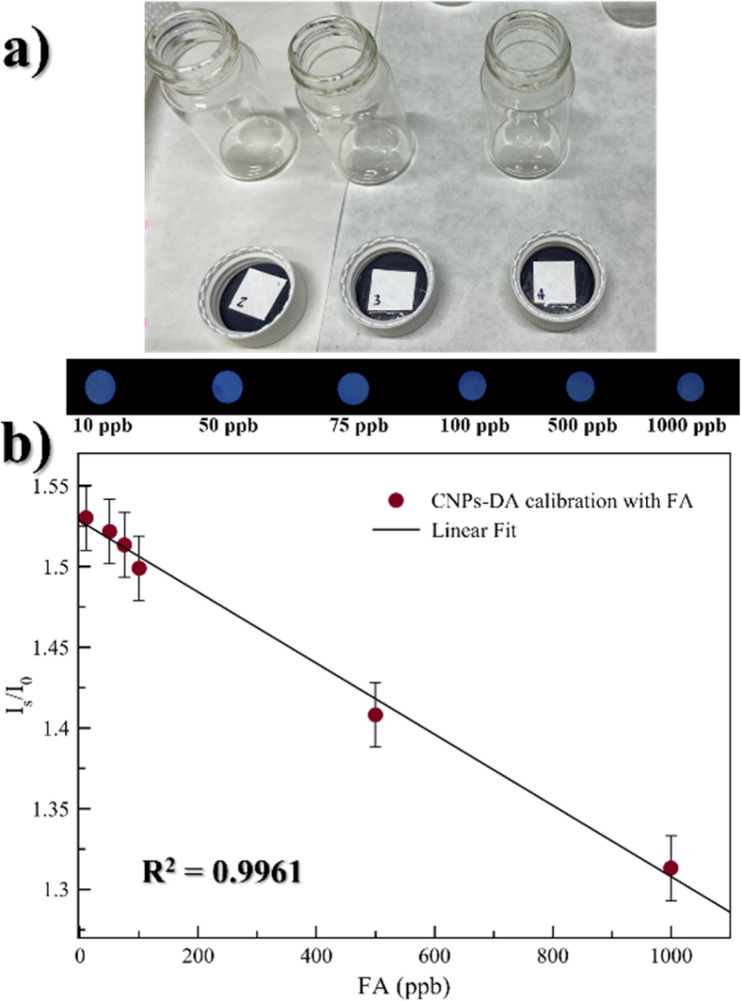
(a) Real image of the experimental setup. (b) Linear range for gaseous FA sensing between 10 ppb and 1 ppm, with *R*^2^ = 0.9961; real images of the spots under a UV LED are reported on the top of the plot.

Calibration of the strip test with gaseous FA was performed using eight different concentrations of FA dissolved in water, each in a separate vial (Fig. 6a). The eight vials generated the following concentration of gaseous FA: 10 ppb, 50 ppb, 75 ppb, 100 ppb, 500 ppb and 1 ppm, respectively. After acquiring the initial image of the sensor in the dark chamber, the vials containing a strip test were sealed. They were kept undisturbed in a thermostatic room at 25 °C for 2 hours, as suggested by the kinetics studies, and then the sensors were photographed again. Proper image processing was performed to extract the intensity values. This procedure allowed us to define a linear response range for gaseous FA exposure between 10 ppb and 1 ppm, with *R*^2^ = 0.9961 (Fig. 6b). The same results were obtained using precise volume of gaseous FA released by a gas permeator (see Fig. S11). The experimental limit of detection was determined to be 10 ppb, which is significantly lower than the WHO threshold for gaseous FA exposure (80 ppb).

A comparative analysis of the recent literature on FA sensing systems, classified according to their sensing mechanism (covalent or supramolecular), reveals significant differences in detection performance and operational ease (Table 1).

**Table 1:** Literature analysis of optical sensors for FA detection

Sensing mechanism	LOD	Detector	Matrix	Ref.
Covalent	0.04 ppb	Naked eye	Solution, air	65
Covalent	0.1 ppb	Naked eye	Solution, air	28
Covalent	2.4 ppb	Naked eye	Solution, air	23
Covalent	1.9 ppb	Smartphone	Air	41
Covalent	0.8 ppb	Smartphone	Solution	60
Covalent	13 ppb	Smartphone	Solution, air	24
Supramolecular	27.41 ppb (sol.) 2.61 ppb (gas)	Fluorimeter	Solution, air	42
Supramolecular	27 ppb	Smartphone	Solution, air	43
Supramolecular	5.83 ppb	Smartphone	Solution	27
Supramolecular	10 ppb	Smartphone	Solution, air	This work

Among covalent-based sensors, a copolymer was very recently reported to demonstrate high sensitivity, with a limit of detection (LOD) as low as 0.04 ppb, using simple visual changes observable by the naked eye over time.^65^ Other notable covalent systems include the pillar[5]arene derivative (LOD 0.1 ppb)^28^ and sodium ligninsulfonate-derived carbon dots (LOD 2.4 ppb),^23^ both operable *via* naked-eye detection.

In terms of user-friendly detectors, several covalent sensors have been successfully integrated with smartphone-based platforms, such as a naphthalamide system (LOD 1.9 ppb),^41^ a functionalized BODIPY (LOD 0.8 ppb),^60^ and hybrid carbon dots (LOD 0.013 ppm),^24^ demonstrating the feasibility of low-cost and portable solutions without compromising detection performance.

In the supramolecular sensing category, a supramolecular organic framework exhibited the best detection performance, with an LOD of 2.61 ppb in the gas phase and 27.41 ppb in solution, as measured by fluorimetric methods.^42^ Regarding ease of use, two supramolecular systems based on polyacrylic acid nanoparticles, (LOD 0.027 ppm)^43^ and metal organic frameworks (LOD 5.83 ppb),^27^ were designed to work with smartphone-based detection, highlighting the growing trend toward user-accessible, field-deployable FA sensing technologies.

These results underscore the importance of balancing sensitivity with detector simplicity when designing FA sensors for practical applications, particularly in settings that require portable, real-time analysis by non-specialized personnel.

In this context, our nanosensor based on supramolecular interaction with FA in air shows excellent performances in terms of sensitivity (10 ppb) and the detection method (smartphone) when compared with other similar systems reported previously.

### Determination of FA in real samples

To evaluate the applicability of the CNPs-DA based sensor to real samples, the determination of FA in a commercial paint sample solution was assessed. After performing a pre-treatment procedure, as detailed in the Experimental section, the standard addition method was applied to quantify the presence of FA by adding 2, 10 and 20 µL of FA 10^−2^ M solution to the sample. The resulting fluorescence responses were represented by plotting the corrected maximum emission of the sensor as a function of the added FA concentration (see Fig. S12 of the SI). Through linear regression analysis, the FA concentration of the sample was found to be 30 nM in the cuvette. Considering the dilution steps performed during sample preparation, this value corresponds to 9 ppm of FA in the original paint.

## Experimental section

### Synthesis and functionalization

#### Synthesis of CNPs-COOH

20 g of citric acid were put in a beaker and heated at 300 °C on a heating plate until caramelization occurred. After cooling to room temperature, 100 mL of a 0.25 M NaOH aqueous solution was added dropwise under magnetic stirring. The resulting solution was centrifuged at 3000 rpm for 30 minutes at room temperature. The supernatant was collected and transferred into a dialysis membrane with a molecular weight cut-off around 12–14 kDa. Dialysis was carried out for 2 days, after which the content of the membranes was dried under vacuum.

#### Synthesis of CNPs-PFPh

275 mg of CNPs-COOH were solubilized in 5 mL of pentafluorophenol (PFPh) and heated to 50 °C under magnetic stirring. Then, 880 mg (5 mmol) of 1-ethyl-3-(3-dimethylaminopropyl)carbodiimide hydrochloride (EDC × HCl) were slowly added. The reaction proceeded under a N_2_ atmosphere for 48 h and then brought to room temperature. The reaction mixture was solubilized with 25 mL of dichloromethane and subsequently underwent liquid–liquid extraction with slightly basic water (pH ∼ 9). Being soluble in dichloromethane, CNPs functionalized with PFPh were collected in a round-bottom flask and dried under vacuum. FT-IR characterization of CNPs-PFPh was performed.

#### Synthesis of CNPs-DA

CNPs-PFPh (770 mg) were solubilized with 20 mL of dry CH_2_Cl_2_. Then, 386 mg of dopamine hydrochloride (2.5 mmol) and 2.2 mL of *N*,*N*-diisopropylethylamine (DIPEA) (12.5 mmol) were added to the solution. The reaction was maintained at room temperature under magnetic stirring and a N_2_ atmosphere. After 4 days, the reaction mixture presents itself as a suspension in CH_2_Cl_2_ and was dried under vacuum. A CH_2_Cl_2_–H_2_O extraction was performed, obtaining a precipitate. It was collected and the solvent was eliminated under strong vacuum. Characterization of CNPs-DA involved AFM, TEM, EDX, ^1^H NMR, FT-IR, UV-vis, fluorescence and XPS. ^1^H NMR (400 MHz, DMSO-*d*_6_) *δ* 6.63 (d, *J* = 8.2 Hz, 1H), 6.57 (s, 1H), 6.43 (d, *J* = 8.2 Hz, 1H), 2.88 (t, *J* = 7.6 Hz, 2H), 2.63 (t, *J* = 7.6 Hz, 2H) ppm.

### Morphological characterization (AFM and TEM)

Morphological characterization was performed using a Nanoscope IIIA-MultiMode atomic force microscope (AFM), Digital Instruments (Santa Barbara, CA, USA), operated in tapping mode. Images were recorded at a scan rate of 1 Hz and 512 × 512 pixels per image using Tap 300 G silicon probes (Budget sensors) mounted on cantilevers with a nominal force constant of 40 N m^−1^ and a resonant frequency of 300 kHz. Samples for AFM were prepared by dispersing minute amounts of CNPs-DA in water (final concentration 0.01 mg mL^−1^) and by rapid drop casting the dispersion onto freshly cleaved mica sheets.

The CNPs were analysed using a 300 kV Field Emission Gun (FEG) TEM equipped with a SuperTwin® lens, providing a point resolution of 1.9 Å and a lattice resolution of 0.19 nm. This instrument operates in both TEM and STEM modes. The microscope also supports spectroscopic analysis *via* energy-dispersive X-ray spectroscopy (EDS). TEM samples were prepared by depositing 20 µL of the CNP suspension onto a copper grid (200 mesh) coated with a carbon film. The droplet was allowed to dry at room temperature for 4 hours.

### XPS characterization

X-ray photoelectron spectra (XPS) were measured at a 45° take-off angle relative to the surface sample holder, with a PHI 5000 Versa Probe II system (ULVAC-PHI, INC., base pressure of the main chamber 1 × 10^−8^ Pa).^46,66^ Samples were deposited on Si substrates and excited with monochromatized Al Kα X-ray radiation using a pass energy of 5.85 eV. The instrumental energy resolution was ≤0.5 eV. The XPS peak intensities were obtained after Shirley's background removal.^46,66^ Spectral calibration was achieved by fixing the Ag 3d_5/2_ peak of a clean sample at 368.3 eV.^67^ The atomic concentration analysis was performed by considering the relevant atomic sensitivity factors. The fitting of some XP spectra was carried out using the XPSPEAK4.1 software, by fitting the spectral profiles with Gaussian envelopes after subtraction of the background. This process involves data refinement, based on the method of the least squares fitting, carried out until there is the highest possible correlation between the experimental spectrum and the theoretical profile. The residual or agreement factor *R*, defined by *R* = [Σ(*F*_obs_ − *F*_calc_)^2^/Σ(*F*_obs_)^2^]^1/2^, after minimization of the function Σ(*F*_obs_ − *F*_calc_)^2^, converged to a value of 0.03.

### FT-IR measurements

FT-IR spectra were obtained using a PerkinElmer Spectrum One Spectrophotometer. Samples were prepared as KBr pellets, using KBr as the solid dispersant medium. Measurements were acquired in transmission over the range 4000–450 cm^−1^, with an average of 16 scans per sample.

### UV-visible measurements

The UV-vis absorption spectrum of CNPs-DA was acquired using a JASCO V-750 UV-vis double-beam spectrophotometer equipped with a 1 cm path-length cell (resolution 0.1 nm). The measurement was carried out in quartz cells, with CNPs-DA's concentration of 0.1 mg mL^−1^ in MilliQ water. The wavelength range where the absorption spectrum was recorded is between 600 and 260 nm.

### Procedure for fluorescence measurements and titration

Fluorescence measurements were performed using a JASCO FP-8550 spectrophotometer, with a resolution of 0.5 nm, at room temperature. The emission was recorded at 90° with respect to the exciting beam line using 2.5 : 5 slit widths for all measurements. Starting from a stock solution of CNPs-DA 1 mg mL^−1^ in MilliQ water, 1 : 10 dilution led to the concentration of 0.1 mg mL^−1^ used for fluorescence measurements and titration. For the screening of fluorescence emission, the *λ*_ex_ was varied in a range from 320 nm to 420 nm. CNPs-DA titration with FA was carried out at *λ*_ex_ = 340 nm. FA was added to the fluorescence cuvette from a 10^−4^ M solution in MilliQ water, in a volume range 2–100 µL. The apparent binding constant log *β* with standard deviation was estimated using the HypSpec software (version 1.1.33),^68^ which derives the constant from spectrophotometric data in solution. HypSpec started with an assumed complex formation scheme and used a least-squares approach to derive the spectra of the complexes and stability constants. The *χ*^2^ test (chi-square) was also applied, in which the residuals should follow a normal distribution. So, if the distribution is approximately normal, the value of *χ*^2^ should be around 12 or less. In all cases, *χ*^2^ values < 10 were obtained from 3 independent measurement sets.^69^ The molar concentration of CNPs-DA used in the analysis was estimated to be 10^−7^ M.† All assumptions and calculations are detailed in the SI. The quantum yields were determined using quinine hemisulfate salt monohydrate as the reference standard.^70^ Measurements were carried out for both CNPs-DA alone and after the addition of 5 ppm FA.

### Selectivity tests

For each interferent, aqueous stock solutions were prepared at a concentration of 10^−2^ M. The water solubility of each interferent is reported in the SI (Table S2). A fixed volume of 2 µL from an interferent stock solution was added to a cuvette, containing a final volume of 2 mL of CNPs-DA + interferent in MilliQ water. This results in an interferent concentration of 10 ppm. The same procedure was repeated for each interferent.

### Computational analysis of the sensing mechanism

*Ab initio* and density functional calculations were performed using the Gaussian09 program package.^71^ Optimization of all involved system was performed at the B3LYP/6-31G(d,p) level of theory. All structures were subjected to a full conformational search to ensure that the absolute minimum was reported. Frequencies were calculated and checked out to make sure that all of them were positive and no imaginary frequencies were present. Gaussview software has been used as a graphic interface to visualize the optimized structures. Zero-point energy (ZPE) was included in each result.

### Strip test for gaseous FA sensing

To gain an accurate estimate of gaseous FA concentration, we referred to literature studies reporting the solubility of gaseous FA in water and the generation of FA vapors from FA aqueous solutions. In fact, due to the complexity of the system, Henry's law can't be used, and proper study is necessary. An equation was obtained from Dong and Dasgupta,^64^ and its empirical nature makes it restricted within the aqueous FA concentration range investigated in the study, from 1.00 × 10^−6^ M to 6.86 × 10^−3^ M. The empirical equation is as follows:[HCHO_aq_] = 10^*x*^[HCHO_g_]^*y*^where *x* = 3.8865 and *y* = 1.065 at a constant temperature of 25 °C. We considered this equation suitable for our experimental setup due to the validation provided by the authors under similar conditions. No influence from pH and salts were found. To convert FA concentration between ppm and atm, we consider that, at atmospheric pressure (*P*_atm_ = 1), the partial pressure is equivalent to FA's molar ratio (*χ*_HCHO_), according to the following equation, which also considers the conversion to ppm:
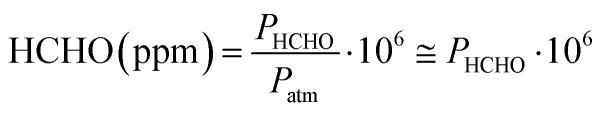


Once the desired FA concentrations in ppm were chosen for calibration of the solid sensor, proper calculations were performed using the two mentioned equations to determine the molar concentration in water. These are shown in the SI.

### Determination of FA in real samples

A commercial wall paint was selected as a real sample for the determination of FA using CNPs-DA in solution. The sample pre-treatment was carried out following the procedure reported by Guo and co-workers.^72^ Specifically, 2 g of paint were mixed with 20 mL of ethanol. After sonication for 20 min and subsequent stirring for 24 h, a precipitate was formed. The supernatant was then filtered through a 0.45 µm filter. The obtained filtrate was analysed by fluorescence spectroscopy using CNPs-DA as the probe. Standard additions of 2, 10 and 20 µL of FA solution (10^−2^ M), respectively, were performed to build the calibration curve. The FA concentration in the sample was determined by linear regression of the fluorescence data and then recalculated to the corresponding concentration in the original paint sample.

## Conclusions

In summary, a novel nanosensor based on carbon nanoparticles functionalized with dopamine was designed and obtained for FA sensing in both aqueous solution and the gas phase, achieving a LOD of 87 ppb and 10 ppb in water and gaseous environments, respectively. The nanoparticle plays a crucial role as an essential component of the sensing mechanism, as supported from computational analysis. The low limits of detection achieved are not merely due to the photophysical properties of the fluorescent core, but rather to the tunable architecture provided by the nanoparticle. In fact, in this system, molecular recognition is not carried out by a single molecular sensor, but it is due to multiple recognition sites anchored to the same nanostructure. This enhances the probability of interaction with the target analyte, allowing for lower sensor concentrations. Our nanosensor can be employed both in water solution and in the solid state for the gaseous FA sensing. Due to the supramolecular interaction between the functionalized nanoparticles and FA, we obtained good selectivity and sensitivity, with LOD values significantly lower than the WHO guideline threshold. The use of a smartphone as the detector leads to the possibility to develop a practical device for real time FA sensing in atmospheric environments. We are working on the development of software to automate the image analyses, and a hardware setup to obtain a commercial kit for FA detection and quantification.

## Author contributions

Conceptualization: N. Tuccitto and G. Trusso Sfrazzetto. Data curation: A. Cavallaro, G. Li-Destri, G. M. L. Consoli, A. Gulino, and A. Pappalardo. Formal analysis: R. Santonocito and M. Petroselli. Investigation: A. Cavallaro, L. Russo, V. Sebastian, R. Ruffino, L. Ferreri, and A. Ferlazzo. Supervision: G. Trusso Sfrazzetto. Writing – original draft preparation: A. Cavallaro and G. Trusso Sfrazzetto.

## Conflicts of interest

There are no conflicts to declare.

## Supplementary Material

NA-008-D5NA00865D-s001

## Data Availability

The data supporting this article have been included as part of the supplementary information (SI). Supplementary information is available. See DOI: https://doi.org/10.1039/d5na00865d.
