# Supplementary material for: Functionalized carbon nanoparticles for smartphone-based sensing of formaldehyde
Source: Nanoscale Adv. 2025 Nov 26;8(1):319–30. doi: 10.1039/d5na00865d (PMC12687286; doi:10.1039/d5na00865d)
Supplement: NA-008-D5NA00865D-s001 [file NA-008-D5NA00865D-s001.pdf]

Supplementary Information (SI) for Nanoscale Advances.
This journal is © The Royal Society of Chemistry 2025

Functionalized Carbon Nanoparticles for Smartphone-Based Sensing of Formaldehyde

Alessia Cavallaro[a], Lorenzo Russo[a], Víctor Sebastián[b], Roberta Ruffino[a,c], Giovanni Li-Destri[a,c], Loredana Ferreri[d], Grazia Maria Letizia Consoli[d], Antonino Gulino[a], Angelo Ferlazzo[a], Andrea Pappalardo[a], Rossella Santonocito[a], Manuel Petroselli[e], Nunzio Tuccitto[a,c] and Giuseppe Trusso Sfrazzetto[a,*]

[a] Department of Chemical Sciences, University of Catania, viale A. Doria 6, 95125, Catania, Italy

[b] Instituto de Nanociencia y Materiales de Aragoń Aragón (INMA), CSIC-Universidad de Zaragoza, Campus Rio Ebro, Edificio I + D + I, C/Poeta Mariano Esquillor, s/n, 50018, Zaragoza, Spain
Department of Chemical and Environmental Engineering, Institute of Nanoscience and Materials of Aragon, Universidad de Zaragoza, Zaragoza, Spain
Networking Research Center in Biomaterials, Bioengineering and Nanomedicine (CIBER-BBN), Instituto de Salud Carlos III, 28029, Madrid, Spain
Laboratorio de Microscopías Avanzadas, Univ. de Zaragoza, 50018, Zaragoza, Spain

[c] CSGI Consorzio Interuniversitario per lo sviluppo dei Sistemi a Grande Interfase, Via della Lastruccia 3, Firenze, Italy

[d] Institute of Biomolecular Chemistry - CNR, Via Paolo Gaifami 18, 95126 Catania, Italy

[e] Department of Science and Technological Innovation, University of Eastern Piedmont "Amedeo Avogadro", Viale Teresa Michel 11, 15121, Alessandria, Italy

[*] Corresponding author. Department of Chemical Sciences, University of Catania, viale A. Doria 6, 95125, Catania, Italy. E-mail address: giuseppe.trusso@unict.it

Supplementary Information

Design of the nanosensor

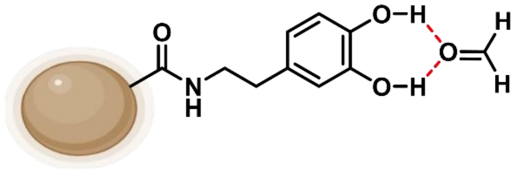

Scheme S1. Schematical representation of the proposed interaction between CNPs-DA and FA.

Structural characterization

- **^{1}H NMR**

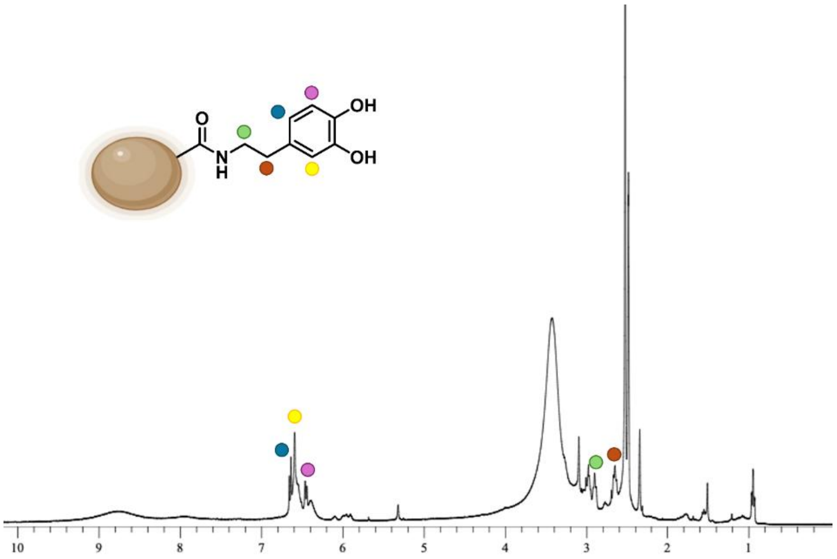

Figure S1. ^{1}H NMR spectrum of CNPs-DA in DMSO-d_6, with attribution of signals.

- **XPS**

Table S1. XPS Binding Energies and assignment.

Atomic state	B. E. (eV)	Assignment
C 1s	284.6<br>285.0<br>285.5<br>286.4<br>288.4 (O=C-N)	sp^2<br>sp^3 C-C, C-H<br>C-N<br>C-OH<br>O=C-N / O=C-
O 1s	531.8<br>533.3<br>533.9	O=C-N<br>C-OH<br>O-Si
N 1s	400.0<br>401.9	O=C-N<br>N^+

- **FT-IR**

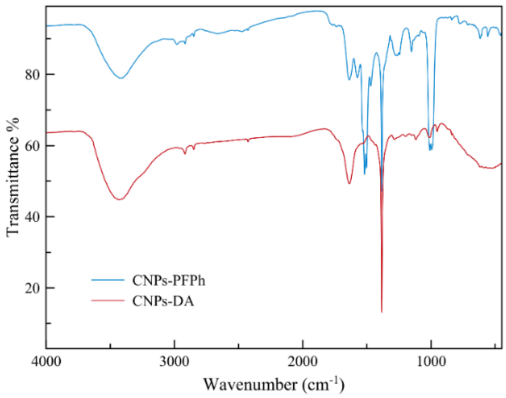

Figure S2. Overlap of the FT-IR spectra of CNPs-PFPh (in blue) and CNPs-DA (in red).

Calculation for CNPs-DA molar concentration

To calculate the molar concentration of the CNPs and then extrapolate the value of the apparent binding constant, some calculations based on theoretical assumptions and experimental results are needed. In particular, AFM results show that the CNPs-DA have a height of approximately 2 nm and a diameter of about 22 nm. For these dimensions, the lateral surface area of a nanoparticle can be estimated to be around 13800 $Å^2$. To estimate the maximum number of dopamine molecules that can be present in the functionalization shell of CNPs. The diameter of a single dopamine molecule was calculated using HyperChem simulations, yielding approximately 5 Å. Considering the molecule as a circle, its surface area is around 20 $Å^2$. The ratio between the lateral surface area of the nanoparticle and the area occupied by a single dopamine molecule suggests that up to 691 dopamine molecules can be present on the surface of one nanoparticle. This is also supported by XPS analysis, which confirms a high degree of surface functionalization.

The following considerations refer to literature data,[1] assuming that the nanoparticle structure can be approximated as stacked graphene sheets. Given the CNPs-DA's diameter, the area of a single sheet of the nanoparticle was calculated to be around 380 nm^2. Graphene is a hexagonal lattice with 0.142 nm between two carbons, which corresponds to the side length of the hexagon and represents the average distance between single (C-C) and double (C=C) bonds. The area of a unit cell in graphene is approximately 0.051 nm^2, which allows us to calculate the density of carbon atoms, knowing that each graphene unit cell contains 2 carbon atoms. This yields a carbon atom density around 39 atoms/nm^2.

Knowing this density, we multiply it for the sheet area of the nanoparticle to obtain approximately 15000 carbon atoms per graphene sheet. Given that the nanoparticle height is 2 nm and the distance between two graphene sheets is 3.4 Å, we estimate that each nanoparticle contains roughly 6 graphene sheets. Therefore, the total number of carbon atoms in one nanoparticle is around 90000, to which we add the contribution from sp^2 carbon atoms coming from dopamine, for a total of about 94200 sp^2 carbon atoms.

These correspond to a molecular weight of approximately 1.13 × 10^{6} atomic mass units. Considering that CNPs-DA concentration in the sensing solution is 0.1 mg mL^{-1}, we obtain a molar concentration in solution of approximately 1 × 10^{-7} M.

The same calculation was repeated multiple times, taking into account the experimental uncertainties on the nanoparticle diameter and height obtained from AFM measurements, as well as considering a lower surface coverage with dopamine (75%). From these further calculations, maximum and minimum molar concentrations were estimated and subsequently used in HypSpec software to determine the binding constants. The obtained variation on the logarithmic constant was 0.08 units, which was taken as the standard deviation of the constant.

HypSpec output file

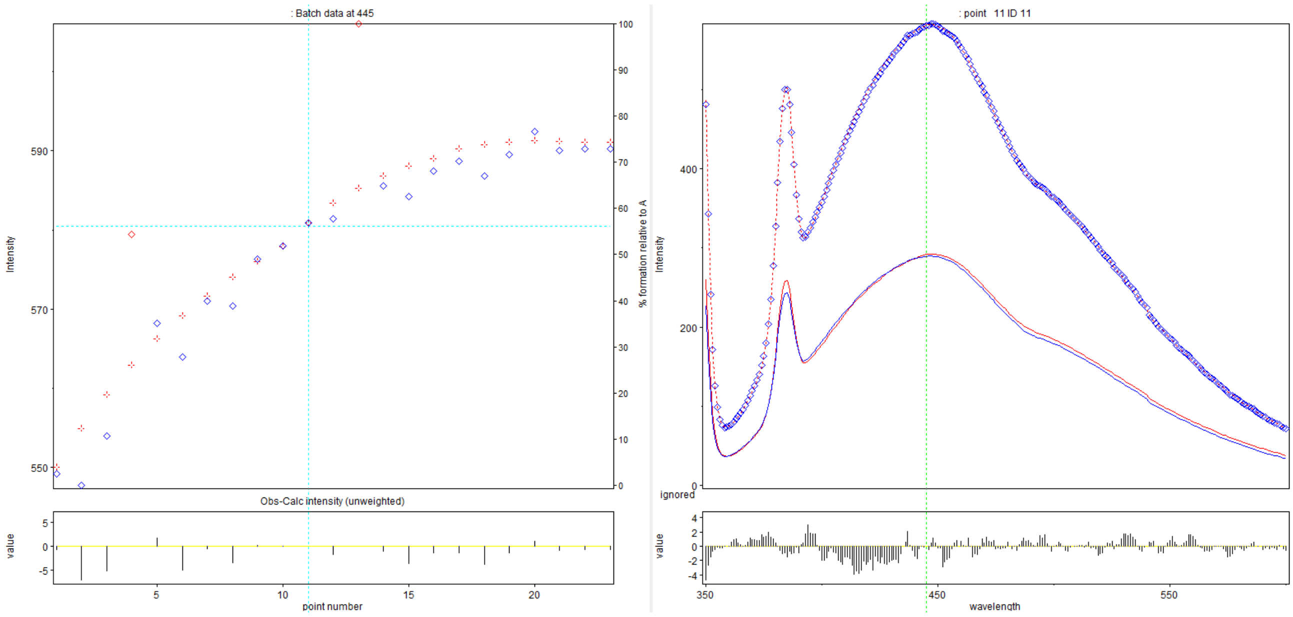


```
                   standard
Log beta   value   deviation
AB         5.869   0.0077
```

Selectivity tests

- **Solubility of the interferents**

  Table S2. Solubility values of each interferent in water at 25°C, according to literature.

**Interferent**	**Solubility (g L^{-1})**	**Ref.**
Acetaldehyde	miscible in all proportions	2
Propionaldehyde	306	
iso-butyraldehyde	56.2	3
Benzaldehyde	6.95	
Methanol	miscible in all proportions	4
Acetone	miscible	5

Ethyl acetate	78.9	6
Toluene	0.556	7

- **Emission spectra of CNPs-DA and CNPs-DA + interferent**

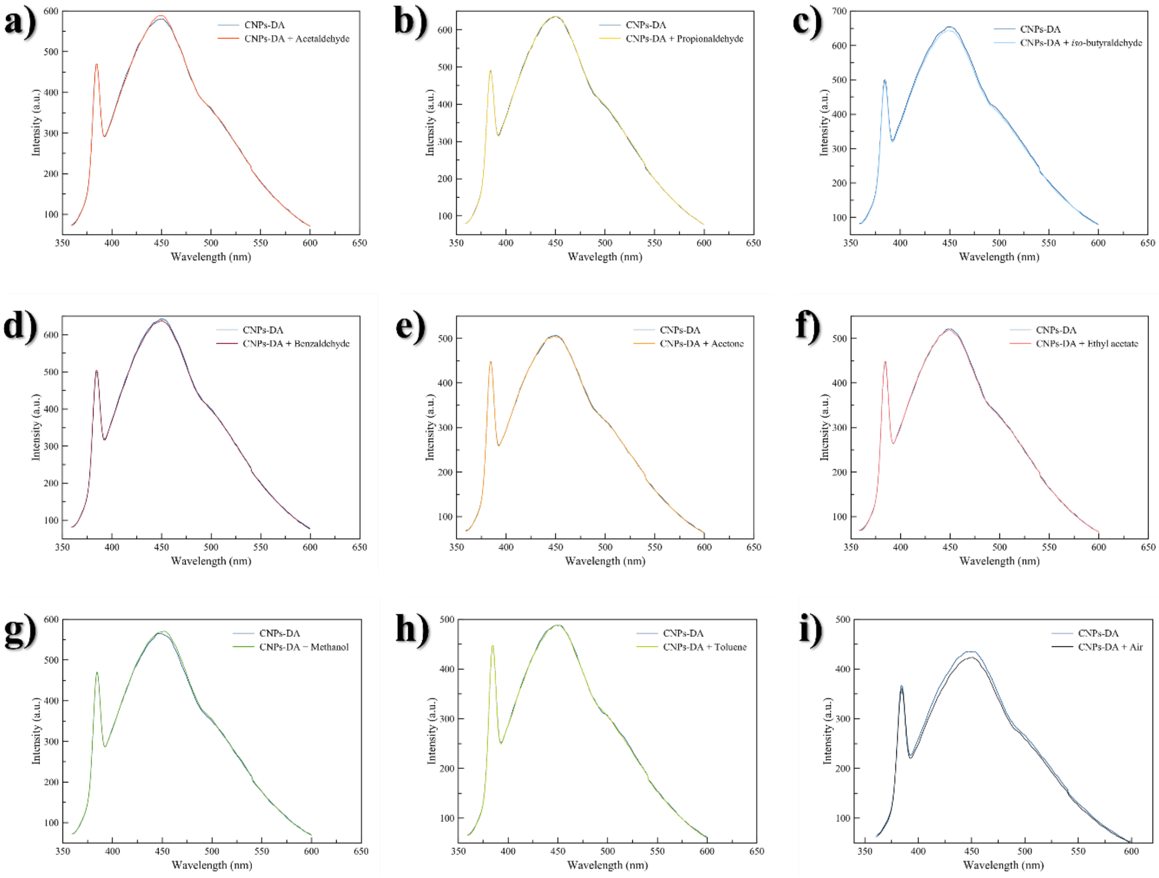

Figure S3. Overlap of CNPs-DA emission spectrum and CNPs-DA + interferent (10 ppm) emission spectrum: a) acetaldehyde, b) propionaldehyde, c) *iso*-butyraldehyde, d) benzaldehyde, e) acetone, f) ethyl acetate, g) methanol, h) toluene and i) air.

Synthesis of CNPs-PEA

CNPs-PFPh (800 mg) were solubilized with 6 mL of dry CH_2Cl_2. 400 mg of 2-Phenylethylamine hydrochloride (2.5 mmol) and 2.21 mL of DIPEA (20.0 mmol) were added to the solution. The reaction mixture was stirred at room temperature under N_2 atmosphere for 4 days. Then, the solvent was removed under vacuum and the solid was solubilized in 20 mL of CH_2Cl_2 and extracted four times with water to remove excesses of the base and the nucleophile. The organic phase was then collected and evaporated under vacuum.

Synthesis of N-Acetyl-Dopamine

To a solution of dopamine hydrochloride (2 g, 10.5 mmol) in a 2:1 mixture of THF (24 mL) and saturated aqueous sodium bicarbonate (12 mL), 1.1 mL of acetic anhydride (11.5 mmol, 1.1 eq.) was added. The mixture was stirred at room temperature for 2 hours and then extracted with ethyl acetate. The organic phase was dried

using anhydrous sodium sulfate and the solvent was removed under reduced pressure. The desired compound was obtained after purification by column chromatography (silica gel, CH_2Cl_2:MeOH 95:5) as a colorless oil. Spectroscopic data were in agreement with those in literature.[8]

Computational analysis of the sensing mechanism

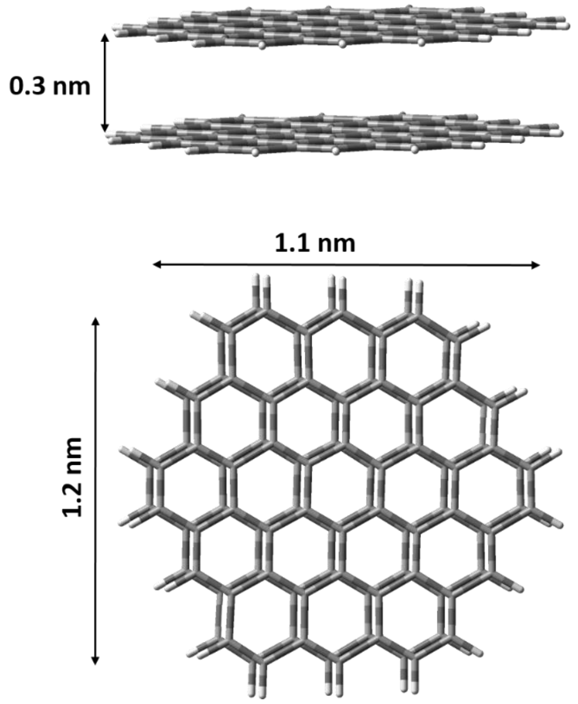

Figure S4. Schematic representation of the unfunctionalized graphene bilayer. Length, size and interspace between the two layers are reported in nm.

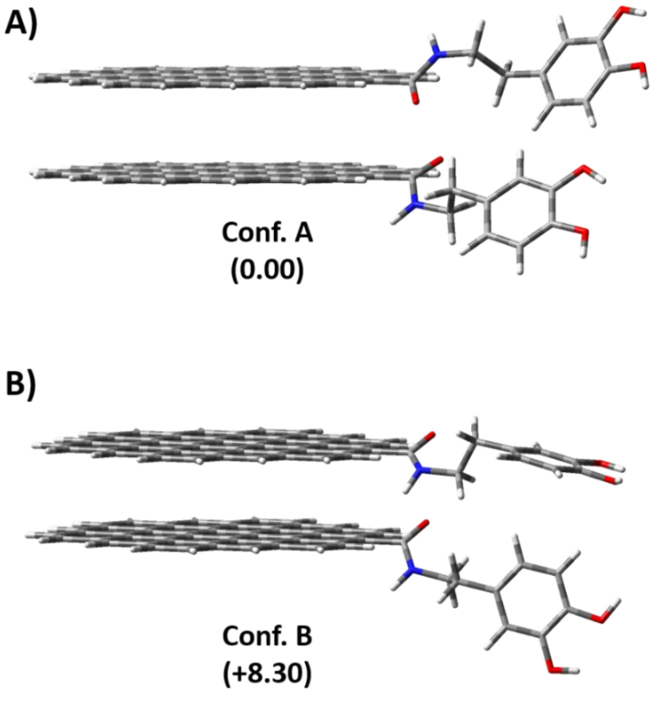

Figure S5. Conformational screening on the CNPs-DA (mCDA) model system at DFT level of theory, taking into account the XPS experimental data. Relative energies (ΔE) are reported between parentheses and expressed in kcal/mol.

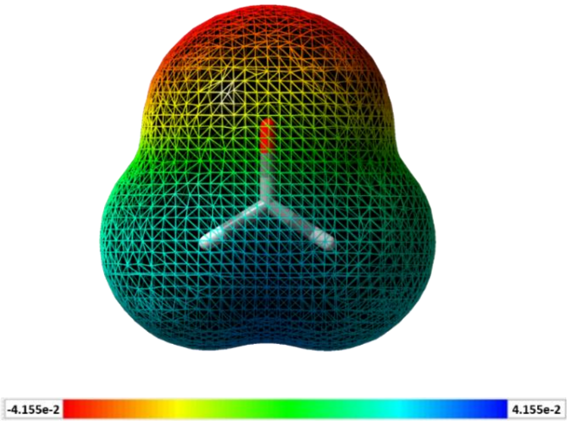

Figure S6. Molecular electrostatic potential (MEP), calculated at DFT level of theory, of the formaldehyde guest.

Table S3. Complexation energy ($E_{complex}$) for FA@CNPs-DA complex calculated at B3LYP/6-31G(d,p) level of theory in the gas phase. Complexation energy of a water dimer ($2H_2O$) is reported for a better comparison. Energy difference (ΔE) is reported, taking into account the FA@CNPs-DA complex as a reference.

**HG Complex**	**$E_{complex}$ (kcal/mol)**	**ΔE (kcal/mol)**
**FA@CNPs-DA**	11.9	-
**$2H_2O$ (dimer)**	5.9	- 6.0

Strip test for gaseous FA sensing

- **Strip test preparation**

  A polyamide filter (pore diameter = 0.2 μm) was chosen as support to realize the solid sensor. It was cut into 1.2 x 1.2 cm squares, optimized to fit inside the cap of a 20 mL vial. Several supports underwent UV/O_3 treatment; then, three drops of CNPs-DA were deposited (1 μL of aqueous solution, 0.5 mg mL^{-1}) onto different areas of each support. The solvent was evaporated under vacuum for 1h, and then with thermic treatment at 80°C for 1h, to be sure all water has been eliminated. Once the sensors returned to room temperature, they were ready for use.

- **Sensing procedure**

  The solid sensors were irradiated with a UV LED (λ_{ex} = 365 nm) inside a 3D-printed dark chamber (Figure S7). The resulting visible emission was photographed with a smartphone, equipped with a 12 megapixels camera. Using the printed box, the distance between the camera and the sensor is always fixed at 20 cm in length. The images were acquired using an application (ProCam®) that allows to lock

different parameters, such as aperture, shutter speed and ISO, to ensure that ambient light does not influence the analyses. An experimental setup was developed by fixing each sensor to the inner surface of the cap of a 20 mL vial. Each system was then sealed and left untouched for the time required to reach the equilibrium, inside a thermostatic room at 25°C. When the equilibrium was reached, according to the time defined by kinetics experiment, the vials were opened, and the sensors were photographed again under UV LED irradiation. The acquired images were processed to extract the analytical results.

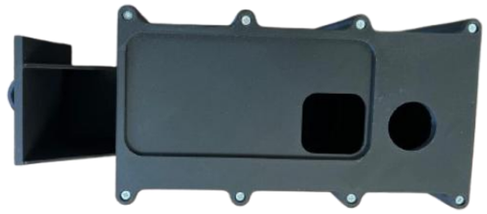

Figure S7. 3D-printed dark chamber with a drawer to insert the solid sensor, a slot for the smartphone and a hole for the UV LED.

- **Images elaboration**

The images were processed using two softwares. The first was Origin and it was used to remove the background contribution from the images. Specifically, a mask was applied to subtract a value of 255 (corresponding to the maximum intensity in the 8-bit grayscale range) from the background, in order to set it to black (0). The fluorescence intensity of the spots remained unchanged. This step was crucial to ensure accurate analysis in the subsequent image processing, performed with the ImageJ software (Figure S8). This allowed us to convert the images colors in RGB channel values, that were then converted into an average in the grey scale value (G), by the application of the formula G = (R_{value} + G_{value} + B_{value})/3. The emission intensity in the greyscale (GI) measured for each spot was compared to the corresponding control value obtained from the sensor before the exposure to FA solution, thus eliminating the UV source drift, according to the following equation:

$$GI_n = \frac{GI_{sample}}{GI_{blank}}$$

With:

GI_n = normalized fluorescence intensity of the sensor

GI_{sample} = fluorescence intensity of the sensor after exposure to gaseous FA

GI_{blank} = fluorescence intensity of the sensor before any exposure.

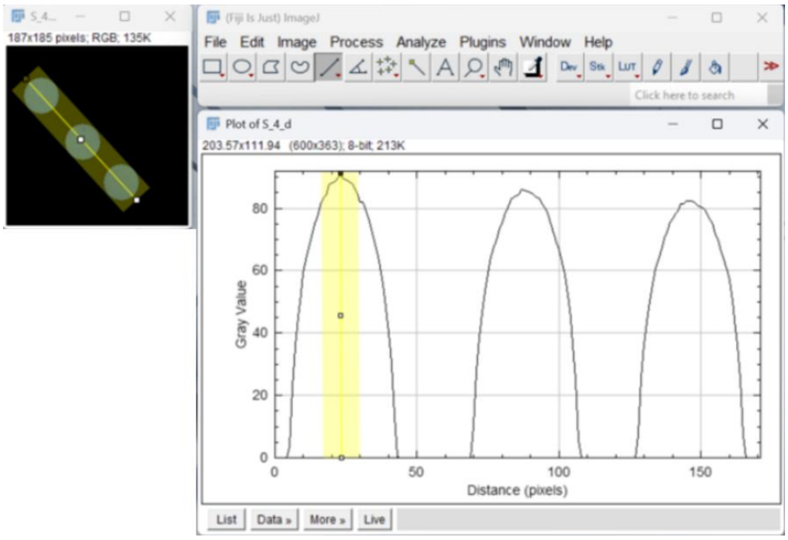

Figure S8. Extrapolation of intensity from image by the program ImageJ.

All the steps described in this section were performed for both kinetic and calibration experiments.

- **Stability of the strip test**

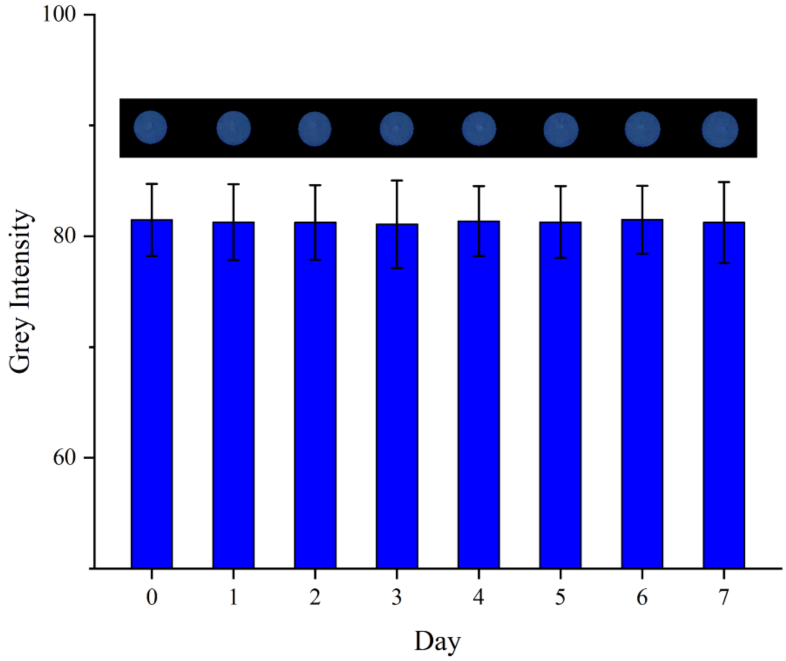

Figure S9. Histogram representing the response of the strip test over a week, once CNPs-DA are drop casted onto the polyamide support. Above each bar, the real image of the sensor under UV irradiation is reported.

- **Calculation of gaseous FA concentration generated from aqueous FA solutions**

1. 1 ppm

   $[HCHO_g] = 1 \cdot 10^{-6}$ atm

   $[HCHO_{aq}] = 10^{3.8865} \cdot (1 \cdot 10^{-6})^{1.055} = 3.60 \cdot 10^{-3}$ M

2. 0.5 ppm

   $[HCHO_g] = 5 \cdot 10^{-7}$ atm

   $[HCHO_{aq}] = 10^{3.8865} \cdot (5 \cdot 10^{-7})^{1.055} = 1.73 \cdot 10^{-3}$ M

3. 0.1 ppm

   $[HCHO_g] = 1 \cdot 10^{-7}$ atm

   $[HCHO_{aq}] = 10^{3.8865} \cdot (1 \cdot 10^{-7})^{1.055} = 3.17 \cdot 10^{-4}$ M

4. 0.075 ppm

   $[HCHO_g] = 7.5 \cdot 10^{-8}$ atm

   $[HCHO_{aq}] = 10^{3.8865} \cdot (7.5 \cdot 10^{-8})^{1.055} = 2.34 \cdot 10^{-4}$ M

5. 0.05 ppm

   $[HCHO_g] = 5 \cdot 10^{-8}$ atm

   $[HCHO_{aq}] = 10^{3.8865} \cdot (5 \cdot 10^{-8})^{1.055} = 1.53 \cdot 10^{-4}$ M

6. 0.01 ppm

   $[HCHO_g] = 1 \cdot 10^{-8}$ atm

   $[HCHO_{aq}] = 10^{3.8865} \cdot (1 \cdot 10^{-8})^{1.055} = 2.80 \cdot 10^{-5}$ M

7. 0.005 ppm

   $[HCHO_g] = 5 \cdot 10^{-9}$ atm

   $[HCHO_{aq}] = 10^{3.8865} \cdot (5 \cdot 10^{-9})^{1.055} = 1.34 \cdot 10^{-5}$ M

8. 0.001 ppm

   $[HCHO_g] = 1 \cdot 10^{-9}$ atm

   $[HCHO_{aq}] = 10^{3.8865} \cdot (1 \cdot 10^{-9})^{1.055} = 2.06 \cdot 10^{-6}$ M

- **Kinetics studies**

  Table S4. Real intensity values obtained from ImageJ elaboration for kinetics studies, as means of three independent measurements.

Sample	Before	After	Ratios
Water 1h	78.97	67.07	0.85
Water 2h	107.87	92.10	0.85
Water 3h	94.57	77.32	0.82
FA 10 ppb 1h	102.56	99.97	0.97
FA 10 ppb 2h	83.51	126.33	1.51
FA10 ppb 3h	76.1	117.53	1.54
FA 1000 ppb 1h	98.30	98.10	1.00
FA 1000 ppb 2h	82.22	113.90	1.38

FA 1000 ppb 3h	73.81	100.27	1.36

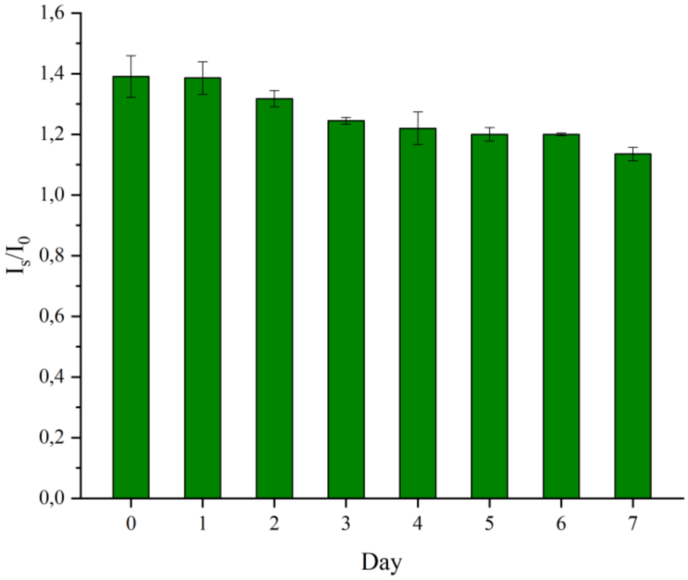

Figure S10. Response of the strip test over a week, after exposure to 1 ppm of gaseous FA in the same experimental conditions to those reported for calibration.

- **Calibration with gaseous FA**

Table S5. Real intensity values obtained from ImageJ elaboration for calibration with gaseous FA, as means of three independent measurements.

**Concentration (ppb)**	**Before**	**After**	**Ratios**
1000	46.83	61.11	1.31
500	45.46	63.46	1.40
100	40.93	61.23	1.49
75	42.65	64.49	1.51
50	59.82	91.15	1.52
10	49.49	75.74	1.53

- **Validation with gas permeator**

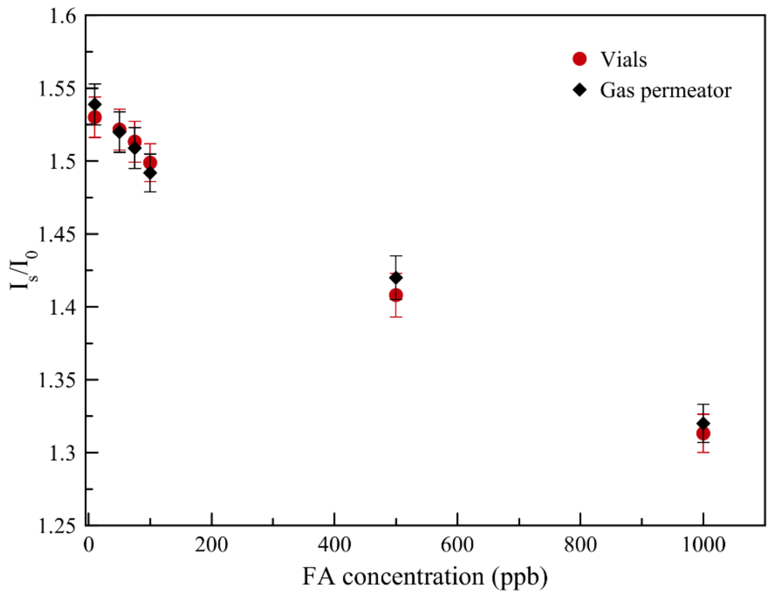

Figure S11. Comparison of the solid CNPs-DA sensor's response to different concentrations of gaseous FA between 10 and 1000 ppb produced from the vials setup (red dots) and the gas permeator (black diamonds), each with relative error bars.

- **Determination of FA in real samples**

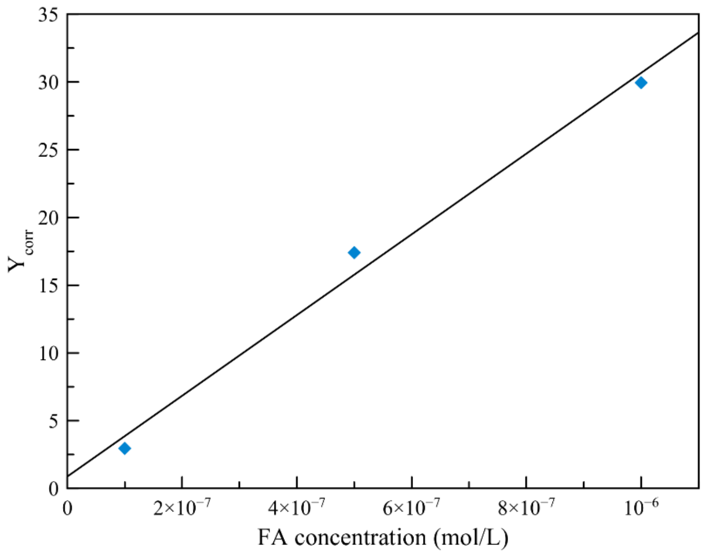

Figure S12. FA quantification in real paint sample: standard additions of 2, 10 and 20 µL of FA 10^{-2} M solution in milliQ water, in the same experimental conditions to those reported for fluorescence titration.

References

1 J.-N. Fuchs and M. O. Goerbig, *Introduction to the Physical Properties of Graphene*, 2008. https://web.physics.ucsb.edu/~phys123B/w2015/pdf_CoursGraphene2008.pdf.

2 T. E. Smith and R. F. Bonner, *Ind. Eng. Chem.*, 1951 **43**, 1169-1173.

3 R. M. Stephenson, *J. Chem. Eng. Data*, 1993, **38**, 630-633.

4 M. Sun, L.-M. Wang, Y. Tian, R. Liu, K. L. Ngai and C. Tan, *J. Phys. Chem. B*, 2011, **115**, 8242-8248.

5 W. M. Haynes, CRC Handbook of Chemistry and Physics, 97th ed., CRC Press in Taylor & Francis Group: Boca Raton, FL, 2017.

6 N. Segatin and C. Klofutar, *Monatshefte für Chemie / Chemical Monthly*, 2001, **132**, 1451-1462.

7 P. Dohányosová, D. Fenclová, P. Vrbka and V. Dohnal, *J. Chem. Eng. Data*, 2001, **46**, 1533-1539.

8 S. H. Kim, T. Söhnel and J. Sperry, *Org. Lett.*, 2020, **22**, 3495-3498.
